# Editorial for the Special Issue on MEMS/NEMS Sensors: Fabrication and Application

**DOI:** 10.3390/mi10090554

**Published:** 2019-08-22

**Authors:** Goutam Koley

**Affiliations:** Department of Electrical and Computer Engineering, Clemson University, Clemson, SC 29634, USA; gkoley@clemson.edu

MEMS sensors are currently undergoing a phase of exciting technological development, not only enabling advancements in traditional applications such as accelerometers and gyroscopes, but also in emerging applications such as microfluidics, thermoelectromechanical, and harsh environment sensors. While traditional MEMS sensors have found wide applications in motion sensing, navigation, and robotics, emerging MEMS sensors are likely to open up applications in the rapidly expanding fields of wearables, internet-of-things, point-of-care detection, and harsh environment monitoring. Novel applications, enabled by advancements in system miniaturization, design innovation and cutting edge fabrication techniques promise an exciting era for MEMS-based sensors and systems development. However, to fully realize their potential several challenges still need to be overcome. Among these challenges, long-term sensor reliability and performance parameter modeling for expedient and robust designs are significant. Additionally, there are issues of cost and power consumption, especially for mass applications requiring small size and weight. 

There are 17 papers published in this Special Issue focusing on a wide range of MEMS sensor applications and fabrication methodologies. Almost a third of the papers, [1,2,3,4,5], and [6], present various accelerometer and gyroscope designs and their performance evaluation. Three of the papers, [7,8], and [9], explore novel fabrication methodologies for MEMS devices. The remaining papers cover various novel MEMS sensors and actuators focusing on inertial micro-switch [10], micro hot plates [11], near IR spectrometry [12], magnetic microactuator [13], resonant microfluidic chip [14], high temperature pressure sensors [15], thermoelectric power sensors [16], and a review of the photonic crystal nanobeams for sensing [17].

In particular, Yang et al. [1] proposed a z-axis magnetoresistive accelerometer with electrostatic force feedback in a three-layer design, taking advantage of the change in a magnetic field caused by input acceleration, which is measured by a pair of magnetoresistive sensors at the top layer. They achieved a good sensitivity of 8.85 mV/g for a plate gap of 1 mm. Qin et al. investigated the effect of anisotropy in single-crystal silicon vibrating ring gyroscope, and found out that the frequency split is much more for the [100] direction compared to [111] direction for n = 2 mode, concluding that fabrication in the latter direction is preferable [2]. Liu et al. presented an ASIC-based design process for a monolithic CMOS MEMS accelerometer [3]. They also presented a low-noise and low zero-g offset design of MEMS accelerometer using a low noise chopper circuit and telescopic architecture, which significantly reduced noise and zero-g offset, but increased the power requirement [4]. Jia et al. addressed an important problem of frequency mismatch in MEMS gyroscopes, and presented an approach for reducing it by designing a dual-mass gyroscope that utilizes a quadrature modulation signal [5]. A maximum frequency mismatch of less than 0.3 Hz was demonstrated using their design. Fang et al. proposed a novel adaptive control algorithm incorporating a back-stepping technique to compensate for model uncertainties, disturbances, and unknown parameters in micro-gyroscopes, which are very pertinent issues in their performance optimization [6].

On the fabrication techniques for the MEMS sensors, Smiljanić et al. reported on the deep wet etching of Si substrate in various crystallographic directions and performed theoretical modeling of the etch profiles, which agreed well with the experimental results [7]. Wu et al. presented an innovative fabrication method for a catalytic gas sensor based on a Pt coil addressing the non-uniformity of pellistor material at the inside surface of the coil [8]. Using a droplet-based coating methodology they demonstrated uniformly coated and reliable pellistor sensors. Kim et al. presented a femtosecond laser-based micro-welding technique for bonding glass and fabricate reliable microfluidic channels. They compared the microfluidic channels fabricated using this method with those fabricated using a glue-based technique, highlighting their relative ease of fabrication and reliability [9].

On the new device applications side, Peng et al. presented an inertial microswitch with a very low threshold of 5 g and high threshold accuracy, leveraging squeeze film damping [10]. Liu et al. presented novel designs of micro hot-plates with significantly improved temperature non-uniformity [11]. Huang et al. reported on a novel MEMS-based infrared spectrometer operating in the range of 800–1800 nm with a wavelength resolution of 10 nm, which compared favorably with similar commercial systems [12]. Feng et al. designed, simulated and fabricated a linear magnetic microactuator with bistable behavior with less than 1 ms response time [13]. An LC resonant circuit-based sensor for detecting metallic debris in hydraulic fuel is proposed by Yu et al., where they were able to successfully demonstrate selective detection of iron and copper particles with diameters down to tens of microns [14]. Gajula et al. designed a GaN circular membrane-based pressure sensor capable of operating at high temperatures. The pressure sensors exhibited high sensitivity at temperatures in excess of 200 °C, which is a significant improvement over their Si counterparts [15]. Zhang et al. presented a MEMS-based thermoelectric power sensor for measuring microwave power using a floating slug design to minimize microwave power loss. The sensor was implemented with GaAs MMIC technology and exhibited very good sensitivity up to 25 GHz [16]. Finally, Qiao et al. presented a comprehensive review of photonic crystal nanobeam-based sensors providing a ready reference for researchers interested in this area. They specifically focused on the sensing of refractive index changes, nanoparticle sensing, optomechanical sensing, and temperature sensing [17]. 

I would like to take this opportunity to thank all the authors for submitting their papers to this Special Issue. I would also like to thank all the reviewers for dedicating their time and helping to improve the quality of the submitted papers.

## References

[1] Yang B., Wang B., Yan H., Gao X. (2019). Design of a Micromachined Z-axis Tunneling Magnetoresistive Accelerometer with Electrostatic Force Feedback. Micromachines.

[2] Qin Z., Gao Y., Jia J., Ding X., Huang L., Li H. (2019). The Effect of the Anisotropy of Single Crystal Silicon on the Frequency Split of Vibrating Ring Gyroscopes. Micromachines.

[3] Liu Y., Wen K. (2019). Implementation of a CMOS/MEMS Accelerometer with ASIC Processes. Mcromachines.

[4] Liu Y., Wen K. (2018). Monolithic Low Noise and Low Zero-g Offset CMOS/MEMS Accelerometer Readout Scheme. Micromachines.

[5] Jia J., Ding X., Gao Y., Li H. (2018). Automatic Frequency Tuning Technology for Dual-Mass MEMS Gyroscope Based on a Quadrature Modulation Signal. Micromachines.

[6] Fang Y., Fei J., Yang Y. (2018). Adaptive Backstepping Design of a Microgyroscope. Micromachines.

[7] Smiljanić M., Lazić Ž., Radjenović B., Radmilović-Radjenović M., Jović V. (2019). Evolution of Si Crystallographic Planes-Etching of Square and Circle Patterns in 25 wt% TMAH. Micromachines.

[8] Wu L., Zhang T., Wang H., Tang C., Zhang L. (2019). A Novel Fabricating Process of Catalytic Gas Sensor Based on Droplet Generating Technology. Micromachines.

[9] Kim S., Kim J., Joung Y., Choi J., Koo C. (2018). Bonding Strength of a Glass Microfluidic Device Fabricated by Femtosecond Laser Micromachining and Direct Welding. Micromachines.

[10] Peng Y., Wu G., Pan C., Lv C., Luo T. (2018). A 5 g Inertial Micro-Switch with Enhanced Threshold Accuracy Using Squeeze-Film Damping. Micromachines.

[11] Liu Q., Ding G., Wang Y., Yao J. (2018). Thermal Performance of Micro Hotplates with Novel Shapes Based on Single-Layer SiO_2_ Suspended Film. Micromachines.

[12] Huang J., Wen Q., Nie Q., Chang F., Zhou Y., Wen Z. (2018). Miniaturized NIR Spectrometer Based on Novel MOEMS Scanning Tilted Grating. Micromachines.

[13] Feng H., Miao X., Yang Z. (2018). Design, Simulation and Experimental Study of the Linear Magnetic Microactuator. Micromachines.

[14] Yu Z., Zeng L., Zhang H., Yang G., Wang W., Zhang W. (2018). Frequency Characteristic of Resonant Micro Fluidic Chip for Oil Detection Based on Resistance Parameter. Micromachines.

[15] Gajula D., Jahangir I., Koley G. (2018). High Temperature AlGaN/GaN Membrane Based Pressure Sensors. Micromachines.

[16] Zhang Z., Ma Y. (2018). DC-25 GHz and Low-Loss MEMS Thermoelectric Power Sensors with Floating Thermal Slug and Reliable Back Cavity Based on GaAs MMIC Technology. Micromachines.

[17] Qiao Q., Xia J., Lee C., Zhou G. (2018). Applications of Photonic Crystal Nanobeam Cavities for Sensing. Micromachines.

